# Complete genome sequence of *Microbacterium* sp. che218, an endophyte isolated from *Vitis vinifera* cv. Chardonnay shoot xylem

**DOI:** 10.1128/mra.00536-24

**Published:** 2024-10-04

**Authors:** Yoshinao Aoki, Takayuki Asada, Masutoshi Nojiri, Shunji Suzuki

**Affiliations:** 1 Laboratory of Fruit Genetic Engineering, The Institute of Enology and Viticulture, University of Yamanashi, Yamanashi, Japan; 2 Agri-Bio Research Center, Kaneka Corporation, Shizuoka, Japan; University of Strathclyde, Glasgow, United Kingdom

**Keywords:** *Microbacterium*, genome sequence, endophytes, grapevine, *Vitis vinifera*, anthocyanin

## Abstract

*Microbacterium* species are bacterial endophytes that live inside plants. We report the complete genome sequence of *Microbacterium* sp. che218, an endophyte isolated from the shoot xylem of the wine grape *Vitis vinifera* cv. Chardonnay.

## ANNOUNCEMENT

To provide new information on the interaction between grapevines and microorganisms, we focused on the endophytic bacteria in grapevine (1). Two Chardonnay grapevines were cultivated in the experimental vineyard of the University of Yamanashi, Japan (latitude 35.6800524, longitude 138.569268). The grapevines were approximately 30 years old and trained to the Guyot-style training system. On 31 December 2019, four first internodes from the bottom of a shoot from the grapevines were surface sterilized with a 0.1% (vol/vol) sodium hypochlorite solution. Bark and epidermal tissues were peeled off from the internodes using a sterilized knife. Xylem tissues were shaved using a sterilized grater. One microgram of the shavings was shaken at 130 rpm in 40 mL of phosphate buffer (pH 7.4) at 25°C for 3 h. After removing xylem debris by filtration through a sterilized cotton gauze, the filtrate was incubated in soybean casein digest (SCD) agar plates at 25°C for 3 days. Restreaking to SCD agar plates was performed twice. From 60 bacterial endophytes on the plates, *Microbacterium* sp. che218 was obtained as a pure culture.

A single colony of che218 was shake-incubated at 160 rpm in an SCD liquid medium at 25°C for 3 days. Genomic DNA was extracted from the cell pellet using Genomic-tip 100/G (Qiagen). Low molecular weight DNA was removed using Short Read Eliminator (PacBio). Furthermore, using the g-TUBE (Covaris), DNA was fragmented to approximately 10–20 kbp. An Ultra-Low DNA input library was prepared using an SMRTbell gDNA Amplification Kit (PacBio) and an SMRTbell Express Template Prep Kit 2.0 (PacBio). After binding the sequencing polymerase to the library using a Revio Polymerase Kit (PacBio), sequencing was performed using Revio (PacBio). Subreads were generated after removing overhang adapter sequences using SMRT Link (PacBio, version 12.0.0.177059). Consensus sequence (CCS) reads were generated by aligning the subreads. CCS reads with an average quality value of less than 20 per read were removed and defined as HiFi reads (31,982 reads). The *N*_50_ for the PacBio read length was 8,913 bp. Ultra-Low PCR adapter sequences were removed from HiFi reads, and PCR duplicate reads were removed using pbmarkdup (PacBio, version 1.0.3). HiFi reads of less than 1,000 bp were deleted using Fitlong (version 0.2.1) (2). Finally, 26,860 reads were obtained, and the total number of bases was 247,494,146. Assembling the HiFi reads using Flye (version 2.9.2-b1786) (3) generated a circular contig, which was rotated at the 16S ribosomal RNA as a start site but not flipped. Assessment by CheckM (version 1.1.2) (4) indicated no contamination and 99.9% completeness of the genome. Default parameters were used for all software.

The circular genome consists of a single contig of 3,591,470 bp. GC content is 70.4%. Annotation by DFAST (version 1.2.0) (5) predicted 3,387 coding sequences, 9 rRNA genes, and 52 tRNA genes. Coding ratio was 90.5%. The average nucleotide identity analysis of che218 with *Microbacterium enclense* isolate bin7 whole genome (CP116226) by ANI Calculator in the EzBioCloud (version 20230823) (6) showed that OrthoANIu value was 82.97%.

## Data Availability

This whole genome shotgun project has been deposited in DDBJ/ENA/GenBank under the accession numbers AP031501 (whole genome), PRJDB17835 (BioProject), SAMD00769561 (BioSample), DRX530437 (Experiment), and DRR546667 (raw sequencing reads).
